# Flaxseed supplementation improved insulin resistance in obese glucose intolerant people: a randomized crossover design

**DOI:** 10.1186/1475-2891-10-44

**Published:** 2011-05-09

**Authors:** Yeong Rhee, Ardith Brunt

**Affiliations:** 1 Department of Health, Nutrition, and Exercise Sciences, North Dakota State University Fargo, ND 58108-6050, USA

**Keywords:** flaxseed, insulin resistance, oxidative stress, inflammation

## Abstract

**Background:**

Obesity leads to an increase in inflammation and insulin resistance. This study determined antioxidant activity of flaxseed and its role in inflammation and insulin resistance in obese glucose intolerant people.

**Methods:**

Using a randomized crossover design, nine obese glucose intolerant people consumed 40 g ground flaxseed or 40 g wheat bran daily for 12 weeks with a 4-week washout period. Plasma inflammation biomarkers (CRP, TNF-α, and IL-6), glucose, insulin, and thiobaribituric acid reactive substance (TBARS) were measured before and after of each supplementation.

**Results:**

Flaxseed supplementation decreased TBARS (p = 0.0215) and HOMA-IR (p = 0.0382). Flaxseed or wheat bran supplementation did not change plasma inflammatory biomarkers. A positive relationship was found between TBARS and HOMA-IR (r = 0.62, p = 0.0003).

**Conclusions:**

The results of the study weakly support that decreased insulin resistance might have been secondary to antioxidant activity of flaxseed. However, the mechanism(s) of decreased insulin resistance by flaxseed should be further determined using flaxseed lignan.

## Background

Obesity is a major public health problem [1]. Obesity increases insulin resistance, reactive oxygen species (ROS) generation and nuclear factor (NF)-κB activation [2,3]. The increase in NF-κB activation leads to low grade inflammation and contributes to the development of diabetes [4]. Studies have reported that pro-inflammatory cytokines, tumor necrosis factor (TNF)-α and interleukin (IL)-6, are associated with an increased hepatic C-reactive protein (CRP) synthesis, inflammation, and insulin resistance in humans and animals [5-14]. TNF-α has been positively related to factors associated with metabolic syndrome, including increased triglyceride concentration, blood pressure, and body mass index (BMI) [5,10].

Antioxidants have been reported to attenuate inflammatory response, insulin resistance, and diabetes development [15-17]. One promising antioxidant is flaxseed. The active ingredient of flaxseed (lignan, secoisolariciresinol diglucoside (SDG)) has significant antioxidant effects by inhibiting DNA scissions and lipid peroxidation and decreaseing ROS [18-21]. Flaxseed also has significant anti-inflammatory effects [22-26]. Flaxseed oil, flaxseed lignan, or flaxseed supplementation significantly decreased serum TNF-α, IL-1 β, IL-6, CRP, glucose, or glycosylated haemoglobin concentrations or increased insulin sensitivity in humans [25-30]. Dietary flaxseed, flaxseed oil, or flaxseed lignan decreased inflammation, oxidative lung damages, lipid peroxidation, or hyperinsulinemia in animals [24,31-33].

Flaxseed is safe and readily available for dietary consumption that positively affects inflammation, glycemic control, and oxidative stress. The current study was conducted to determine the mechanism(s) by which flaxseed offers the protection against insulin resistance and inflammation via regulation of oxidative stress. It was hypothesized that flaxseed supplementation will decrease oxidative stress, thus reducing inflammation biomarkers and insulin resistance. The effects of flaxseed on oxidative stress, insulin resistance, and inflammation were reported in this article.

## Methods

The present study was approved by the Institutional Review Board at North Dakota State University. Written consent was obtained from all participants before the initiation of the study.

### Participant Selection

Potential participants who were overweight, hypertensive, and with a family history of diabetes were screened for impaired fasting plasma glucose. Following a positive impaired fasting plasma glucose (>100 mg/dL), an oral glucose tolerance test (OGTT) was completed. Individuals included into the study had fasting plasma glucose concentration between 100-125 mg/dL, and following a 100 g oral glucose load, had 2-hour plasma glucose of greater than 140 mg/dL but less than 199 mg/dL. The dose of 100 g oral glucose was used following current diagnostic test guidelines of Sanford Health Laboratory in consideration of obesity and increased body surface area to be covered by glucose solution [34]. General health status including vitamin/mineral/herbal supplementation and medication intake was determined by health questionnaires before and at the end of the study. Individuals excluded from the study included: people (1) on prescribed oral hypoglycemic medication or insulin injection; (2) had any diagnosed illness other than controlled hypertension or impaired glucose tolerance; or (3) allergic to either flaxseed or wheat. Thirty three people were screened and 11 qualified for study participation.

### Design and Treatments

A randomized crossover research design was used for the study. Eleven participants were randomly assigned into one of two groups: flaxseed or wheat bran supplementation group. Participants received a daily allotment of either 40 g of wheat bran or flaxseed in form of ground grain or bread for 12 weeks. Participants were instructed to incorporate the supplement in their daily meals using a method of their choice. Following a 4-week washout period of no supplements, participants received the alternate supplement (either flaxseed or wheat bran) for another 12 weeks. The ingredients in the bread were adjusted to provide similar calories in both supplemental breads. The bread was baked at a local bakery. Before entering the study, participants were instructed to substitute the study supplement for some of their usual carbohydrate and fat consumed at the meal so that no additional calories would be consumed. Participants were requested to keep the supplement in the freezer to maintain freshness. Participants were also asked to record the amount eaten and to return leftover supplements to the investigators. The collected leftover supplements were weighed by the investigators to check dietary compliance. Nutrient compositions of wheat bran bread or flaxseed bread were analyzed using Food Processor software (version 10.5.1, ESHA Research, Salem, OR).

A health questionnaire that included birth date, current health conditions, medication use, vitamin, mineral, or herbal supplement use, and exercise practices was completed at the screening. A follow-up health questionnaire was completed at the end of the study to determine if any changes had occurred in the participant's health or exercise practices. Each participant's height (without shoes) was measured during the screening test. Using a balance beam scale, each participant was weighed at the screening, at the beginning and end of each supplementation. BMI was calculated using the equation BMI = weight (kg)/height^2 ^(m^2^). Waist circumference was measured using a measuring tape. The measuring tape was placed around the abdomen at the level of the iliac crest and took the reading at the end of a normal expiration [35]. The Block Brief 2000 Food Questionnaire, a validated self-administered 66-item semi-quantitative food frequency questionnaire [36], was administered at the beginning of each supplementation.

### Blood Sample Collection and Laboratory Assays

After a 12-hour fast, participants had venous blood drawn. Samples were collected before and after each 12-week supplementation. Fasting plasma glucose concentration was measured by Sanford Health; the result was reported elsewhere [37]. Plasma insulin concentration was measured using an enzyme-linked immunosorbent assay (ELISA) kit (Alpco Co., Salem, NH). Plasma TNF-α (Invitrogen, Carlsbad, CA), IL-6 (Cayman Chemical, Ann Arbor, MI), and high sensitivity (hs)-CRP (BioCheck, Inc., Burlingame, CA) concentrations were measured using ELISA kits. Thiobaribituric acid reactive substance (TBARS) was measured to estimate oxidative stress using a kit (ZeptoMetrix Co, Buffalo, NY). Homeostasis model assessment (HOMA-IR) was used to estimate insulin resistance. The following formula was used for HOMA-IR calculation: (insulin [pmol/L] × glucose [mmol/L]/22.5) [38].

### Statistical Analysis

SAS software (version 9.1; SAS, Cary, NC) was used for data analysis. Data were tested for normality by a univariate test and homogeneity of variances by a t-test. The effect of treatment groups (between wheat bran and flaxseed groups) on plasma insulin, HOMA-IR, TBARS, TNF-α, IL-6, and hs-CRP concentrations was assessed using two sample t-tests whereas the effect of the 12 weeks of supplementation (within each supplementation group) on plasma insulin, HOMA-IR, TBARS, TNF-α, IL-6, and hs-CRP concentrations was assessed using paired t-tests. Pearson correlation coefficients were used to assess relationships among the test variables. A type I error rate of 0.05 was used to assess significance for all statistical tests.

## Results

A total of eleven people initially participated in the study. Four participants dropped out: two after first blood sample collection and another two after the third blood sample collection. The first two participants withdrew from the study due to personal reasons unrelated to the study; the latter two participants dropped out of the study because they did not like the wheat bran supplement. The two who dropped out during the first phase of supplementation were excluded from the data analysis; however, the participants who dropped out during the second phase of supplementation were included in the data analysis. The overall compliance rate with dietary supplements was >95%. Supplemental bread that contained 40 g of either ground flaxseed or wheat bran, provided 453 kcal and 398 kcal, respectively [37]. Table 1 shows the nutrient composition of supplemental bread.

**Table 1:** Composition of daily supplemental flaxseed bread and wheat bran bread*

Measures	Flaxseed	Wheat bran
Energy (kcal/d)	453	398
Carbohydrate (g/d)	72.2	83.4
Fiber (g/d)	13.1	18.6
Protein (g/d)	16.8	14.8
Fat (g/d)	16.4	6.0
Saturated fatty acids (g/d)	6.6	0.8
Monounsaturated fatty acids (g/d)	4.5	1.3
Polyunsaturated fatty acids (g/d)	5.0	3.2
Omega 3 fatty acid (g/d)	8.4	0.1
Omega 6 fatty acid (g/d)	2.5	2.9

*Composition of flaxseed and wheat bran bread was reported previously [37].

Composition of daily supplemental flaxseed bread and wheat bran bread are based on ingredient analysis using Food Processor Software (version 10.5.1, ESHA Research, Salem, OR).

The average age of the participants was 54.7 ± 6.6 years. Of the nine participants, four were male and five were female. Participants reported no change in dietary intake, health status including inflammatory status, or exercise habits throughout the study. All participants were nonsmokers and reported exercising for 30 minutes or more per day, 3-5 days per week. Six participants reported taking multivitamins; two participants reported taking calcium/magnesium supplements; and one participant reported taking a vitamin E supplement.

Body weight and BMI were not significantly different compared to the baseline, nor between treatment groups. The average body weight was 96.2 ± 18.9 vs. 92.2 ± 20.4 (kg), flaxseed group; 94.7 ± 20.0 vs. 95.2 ± 20.3 (kg), wheat bran group at the baseline and end of the study, respectively. The average BMI was 32.4 ± 8.2 vs. 30.3 ± 5.5 (kg/m^2^), flaxseed group; 32.0 ± 8.3 vs. 32.4 ± 8.3 (kg/m^2^), wheat bran group at the baseline and end of the study, respectively. No significant relationships between weight change and glucose, insulin, HOMA-IR, or inflammatory biomarkers were found. The waist circumference did not change significantly. The average waist circumference was 106.9 ± 13.5 vs. 105.9 ± 12.7 (cm), flaxseed group; 108.7 ± 12.2 vs. 106.7 ± 13.5 (cm), wheat bran group at the baseline and end of the study, respectively.

Based on a self-administered 66-item semi-quantitative food frequency questionnaire, no significant differences were observed in mean daily energy and nutrient intakes between each supplementation. At the beginning of the first supplementation, mean energy intake was 1875 ± 798 kcal/d, with 17%, 38%, and 45% coming from protein, fat and carbohydrates, respectively. At the beginning of the second supplementation, mean energy intake was 2038 ± 973 kcal/d, with 17%, 37%, and 46% coming from protein, fat and carbohydrates, respectively. Vitamins C and E and beta carotene, antioxidants, consumed as food were not significantly different among the participants. Software limitations prevented analysis of the dietary intake of α-linolenic acid and insoluble and soluble fiber.

**Figure 1:**
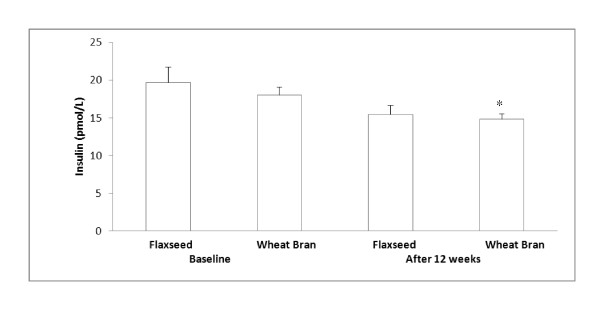
**Fasting plasma insulin concentrations before and after each supplementation**. Data are presented as mean ± standard error, n = 7-9; Statistical significance level was set at 0.05. *Significantly decreased after 12 weeks of wheat bran supplementation compared to the baseline, p = 0.026. No significant change in plasma insulin concentration after 12 weeks of flaxseed supplementation. No significant differences in plasma insulin concentration between flaxseed and wheat bran supplementation groups

Flaxseed supplementation significantly reduced fasting plasma glucose concentration compared to the baseline (0 wk) and compared to the wheat bran supplementation: 5.92 ± 0.31 vs 4.79 ± 0.17 (mmol/L), flaxseed group; 5.67 ± 0.14 vs. 5.73 ± 0.35 (mmol/L), wheat bran group [37]. Flaxseed supplementation did not significantly change fasting plasma insulin concentration (Figure 1). However, 12 weeks of wheat bran supplementation significantly reduced plasma insulin concentration compared to baseline (p = 0.026, Figure 1).

**Figure 2:**
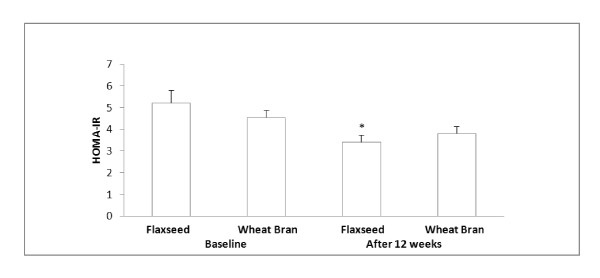
**HOMA-IR index before and after each supplementation**. Data are presented as mean ± standard error, n = 7-9; Statistical significance level was set at 0.05. *Significantly decreased after 12 weeks of flaxseed supplementation compared to the baseline, p = 0.0382. No significant change in HOMA-IR after 12 weeks of wheat bran supplementation. No significant differences in HOMA-IR between flaxseed and wheat bran supplementation groups

**Figure 3:**
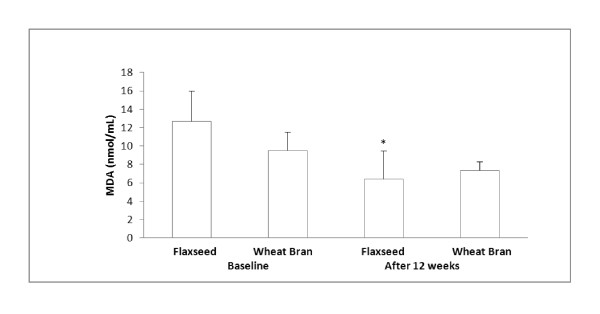
**Plasma TBARS concentrations before and after each supplementation^**1**^**. ^**1**^TBARS is an indicator for lipid peroxidation, and it is measured in MDA equivalents. Data are presented as mean ± standard error, n = 7-9; Statistical significance level was set at 0.05. *Significantly decreased after 12 weeks of flaxseed supplementation compared to the baseline, p = 0.0215. No significant change in plasma TBARS concentration after 12 weeks of wheat bran supplementation. No significant differences in plasma TBARS concentration between flaxseed and wheat bran supplementation groups

When estimating insulin resistance using HOMA-IR [38], flaxseed supplementation significantly decreased an HOMA-IR index (34.7%) compared to the baseline. Wheat bran supplementation did not significantly change an HOMA-IR index (Figure 2). Moreover, flaxseed supplementation decreased plasma TBARS concentration compared to baseline (Figure 3). Significant relationships were found between HOMA-IR and insulin (r = 0.89, p < 0.0001) and HOMA-IR and glucose (r = 0.66, p < 0.0001).

The inflammation biomarkers, TNF-α, IL-6, and hs-CRP did not change significantly following 12 weeks of flaxseed or wheat bran supplementation (Table 2). However, positive relationships between plasma glucose and TBRAS (r = 0.39, p = 0.034), TBARS and TNF-α (r = 0.57, p = 0.0011), TBARS and insulin (r = 0.52, p = 0.0035), and TBARS and HOMA-IR (r = 0.62, p = 0.0003) were found.

**Table 2:** Inflammation biomarker changes over the course of the experiment^1^

	Baseline		After 12 weeks	
	
	Flaxseed	Wheat Bran	Flaxseed	Wheat Bran
IL-6 (pg/mL)	3.2 ± 0.6	3.1 ± 0.5	3.2 ± 0.5	3.6 ± 0.8
TNF-α (pg/mL)	0.9 ± 0.4	0.6 ± 0.5	0.8 ± 0.5	0.7 ± 0.6
hs-CRP (mg/L)	3.6 ± 1.7	4.6 ± 2.1	3.9 ± 0.9	7.2 ± 5.5

^1 ^All values are mean ± standard deviation; n = 7-9; Statistical significance level was set at 0.05

The inflammation biomarkers, IL-6, TNF-α, and hs-CRP did not change significantly following 12 weeks of flaxseed or wheat bran supplementation. No significant difference in IL-6, TNF-α, and hs-CRP between flaxseed and wheat bran supplementation groups

## Discussion

The current study was intended to determine, if the flaxseed supplementation would have an effect on insulin resistance via regulation of oxidative stress in obese glucose intolerant people. The current study found that flaxseed supplementation decreased insulin resistance. Although the plasma insulin concentration did not change significantly, an HOMA-IR index significantly decreased, suggesting a decrease in insulin resistance or decreased glucose concentration following flaxseed supplementation. A highly significant positive relationship (r = 0.89) between HOMA-IR and insulin indicates that the decreased HOMA-IR is more closely related to insulin concentration than glucose concentration. Decreased HOMA-IR following flaxseed lignan or flaxseed supplementation without significant changes in insulin concentration has also been reported by others [28,30]. No significant changes in plasma insulin concentration following flaxseed supplementation might be related to small sample sizes in the current study which warrant the need of future study with a larger sample size.

In the present study, flaxseed supplementation showed a beneficial effect on oxidative stress as determined by TBARS. Flaxseed supplementation significantly decreased TBARS concentration which suggests decreased lipid peroxidation in these participants and an antioxidant activity of flaxseed.

In the present study, TNF-α, IL-6, and CRP remained within normal ranges. The results suggest that these obese participants did not have low grade systemic inflammation, but these participants are classified as a high cardiovascular disease risk group [39]. Studies show that CRP concentration remained the same following flaxseed or flaxseed lignan supplementation while CRP concentration in the control supplementation group increased [29,30,40,41]. A similar result was found in the present study. While CRP concentration in flaxseed supplementation group remained the same as the baseline, it increased following wheat bran (control) supplementation. Although flaxseed oil has been reported to significantly decrease serum TNF-α or IL-6 in humans [25,26], no significant changes were found in TNF-α or IL-6 following flaxseed or flaxseed lignan supplementation [28-30,40] which is in agreement with the findings of the present study.

Increased glucose oxidation and NADPH oxidase activity secondary to hyperglycemia and obesity increase ROS generation [42,43]. Participants in the present study were hyperglycemic and obese, and they had normal TNF-α and IL-6 concentrations which suggest that the participants might have had increased oxidative stress secondary to high plasma glucose concentrations and their obesity not secondary to inflammation. Increased oxidative stress may suppress insulin receptor activation or decrease the translocation of GLUT4 on the cell membrane [44]. It was found that antioxidants increase glucose disposal via increased translocation of GLUT 4 on the cell membrane and increase basal glucose uptake via redistribution of GLUT 1 [44,45]. Although GLUT expression or cellular glucose uptake was not measured in the present study, SDG treatment increased basal glucose uptake in human RBCs in our previous study [46].

Increased ROS, especially hydroxyl radicals increase TBARS concentration. TBARS is an indicator for lipid peroxidation, and it is measured in malondialdehyde (MDA) equivalents. Decreased TBARS concentration following flaxseed supplementation indicates decreased lipid peroxidation. SDG in flaxseed decreases lipid peroxidation by scavenging hydroxyl radical [47,48]. Reduced lipid peroxidation may have maintained cell membrane integrity keeping insulin receptor intact, thus may have contributed to increased glucose disposal. The positive relationships seen between TBARS and insulin and TBARS and HOMA-IR in the present study are in agreement with other study findings that increased ROS triggers insulin resistance [49-52].

Weight loss improves insulin resistance by increasing insulin sensitivity [53]. However, no significant weight changes in these participants suggest that decreased HOMA-IR index is not related to weight loss. Our results support that high glucose concentrations increase oxidative stress [54] as shown increased TBARS. As seen by others [51] the positive relationships between TBARS and HOMA-IR indicate that increased oxidative stress may have increased insulin resistance in these obese participants. Moreover, positive relationships between TBARS and TNF-α concentrations found in this study support that increased oxidative stress increases pro-inflammatory cytokines [55].

Soluble and insoluble fiber in flaxseed shown to improve glycemic control [56], and is inversely related to CRP concentrations [57]. The current study did not evaluate the fiber effects on the insulin resistance and inflammation biomarkers. However, as discussed in elsewhere [37], the wheat bran supplementation provided higher concentration of dietary fiber (18.6 g/d) compared to flaxseed (13.1 g/d) in the current study. In addition, daily dietary fiber intake was not different between flaxseed and wheat bran groups [37]. No significant changes in inflammation biomarkers or insulin resistance indicators (glucose or HOMA-IR) following wheat bran supplementation indicate that the antioxidant activity of flaxseed or other bioactive component of flaxseed such as α-liniolenic acid might have attributed to decreased insulin resistance rather than dietary fiber.

Decreased insulin resistance might be attributed to lignan via its antioxidant activity of flaxseed in these obese participants. However, the present study was not able to identify the direct role of lignan in insulin resistance due to a high α-linolenic acid content in whole flaxseed. Therefore, further study is needed to determine the effects of flaxseed lignan on insulin resistance and peripheral glucose uptake to identify the mechanism(s) of decreased insulin resistance. Although flaxseed decreased HOMA-IR and TBARS, but none of the changes in TBARS, insulin, and HOMA-IR between flaxseed and wheat bran supplementation groups was significantly different. No significant difference in HOMA-IR, insulin, and TBARS between flaxseed and wheat bran supplementation also supports the need of study using flaxseed lignan to further determine the mechanism(s) of decreased insulin resistance via antioxidant activity of flaxseed.

The present results indicate that obese participants were glucose intolerant without low grade systemic inflammation. Since these participants did not have an identified inflammatory condition, flaxseed supplementation may have not affected these inflammation biomarkers. If these participants had systemic inflammation secondary to obesity or impaired glucose tolerance, flaxseed effects on inflammation may have been seen. This also suggests the need of future study using either animal models with inflammation or people with systemic inflammation to determine flaxseed effects on inflammation.

## Conclusions

In conclusion, it was hypothesized that an antioxidant (lignan) in flaxseed would decrease ROS generation which downregulates TNF-α and IL-6 production. Therefore, the bioactive component of flaxseed, lignan, would decrease inflammation and insulin resistance. Although no statistical differences were seen in inflammation biomarkers following flaxseed supplementation, the positive relationship between TBARS and TNF-α suggests that increased oxidative stress would increase TNF-α. Moreover, the positive relationship between TBARS and HOMA-IR suggests that increased oxidative stress increased insulin resistance. Although flaxseed supplementation decreased HOMA-IR and TBRAS independently, these variables were not significantly different compared to wheat bran supplementation. No significant difference in HOMA-IR and TABRS between flaxseed and wheat bran supplementation groups weakly supports a hypothesis of the current study: flaxseed supplementation will decrease oxidative stress, thus reducing inflammation biomarkers and insulin resistance. A small sample size and a cumulative effect of α-linolenic acid and/or fiber in the flaxseed might have caused non-significant differences in HOMA-IR and TBARS between flaxseed and wheat bran supplementation groups. Therefore, further study is needed to determine the role of antioxidant activity of flaxseed in insulin resistance using flaxseed lignan and with a larger sample size.

## List of abbreviations used

BMI: body mass index; ELISA: enzyme-linked immunosorbent assays; HOMA-IR: homeostasis model assessment-insulin resistance; hs-CRP: high sensitivity C-reactive protein; IL: interleukin; MDA: malondialdehyde; NF: nuclear factor; ROS: reactive oxygen species; SDG: secoisolariciresinol diglucoside; TBARS: thiobaribituric acid reactive substance; TNF: tumor necrosis factor.

## Competing interests

The authors declare that they have no competing interests.

## Authors' contributions

YR participated in the design of the study, performed the sample and data analyses, and drafted the manuscript. AB participated in the design of the study and helped draft the manuscript. All authors read and approved the final manuscript.
